# Intranasal midazolam as an alternative to rectal diazepam for management of canine status epilepticus

**DOI:** 10.18849/ve.v9i4.689

**Published:** 2024-12-10

**Authors:** Flora Foxx

**Affiliations:** Emergency Vets 24/7, 124 Anderson Street, Manunda, Queensland, Australia, Australia

**Keywords:** benzodiazepines, canine, epilespy, intranasal midazolam, rectal diazepam, seizures, status epilepticus

## Abstract

**PICO question:**

In canine patients in status epilepticus, is intranasal midazolam as effective as rectal diazepam for controlling seizures?

**Clinical bottom line:**

**Category of research:**

Treatment.

**Number and type of study designs reviewed:**

One study was found—a multicentre randomised open-label clinical trial comparing intranasal midazolam and rectal diazepam in canine patients.

**Strength of evidence:**

Weak.

**Outcomes reported:**

The study critically appraised in this summary indicated that intranasal midazolam is effective in achieving seizure control in canine patients, with tolerable safety margins. Mild adverse effects associated with benzodiazepine administration such as sedation and drowsiness were noted, but there were no reports of serious adverse events related to the use of intranasal midazolam.

**Conclusion:**

Intranasal midazolam appears to be safe and effective for achieving seizure control in canine patients, both in terms of efficacy and speed of onset, and is a suitable first-line treatment option for status epilepticus, especially when intravenous access is not rapidly available. Patients should be monitored after drug administration for development of adverse effects, including sedation, sneezing, and nasal irritation. A nasal mucosal atomisation device may be used to enhance bioavailability of the drug, improving efficacy.

## How to apply this evidence in practice

The application of evidence into practice should take into account multiple factors, not limited to: individual clinical expertise, patient’s circumstances and owners’ values, country, location or clinic where you work, the individual case in front of you, the availability of therapies and resources.

Knowledge Summaries are a resource to help reinforce or inform decision-making. They do not override the responsibility or judgement of the practitioner to do what is best for the animal in their care.

## Clinical scenario

A 4-year-old male neutered border collie previously diagnosed with idiopathic epilepsy presents to your practice in status epilepticus. Due to the severity of the seizure activity, you are unable to successfully place an intravenous catheter and must use another administration route to deliver emergency drugs. Would you choose intranasal midazolam or rectal diazepam as a first-line treatment to achieve cessation of the seizure activity?

## The evidence

Literature searches identified only one paper directly comparing intranasal midazolam (IN-MDZ) and rectal diazepam (R-DZP) in canine patients experiencing status epilepticus (Charalambous, et al., 2017); this was a randomised multi-centre clinical trial.

Charalambous et al. (2017) found that IN-MDZ had a significantly higher success rate than R-DZP, with more rapid seizure control and fewer instances of seizure recurrence in canine patients. Whilst results from this study are promising, there were only 35 dogs included in the trial, reducing the overall statistical power. Furthermore, the researchers were not blinded to the treatment received by each dog, increasing the risk of observer bias when reporting data, especially when making subjective observations, such as whether tremors or other abnormal post-ictal behaviours count as new seizure activity (Hróbjartsson, et al., 2013).

Overall, the appraised study suggests that IN-MDZ is a safe and effective first-line treatment for status epilepticus in canine patients and may be a beneficial alternative to rectal diazepam in cases where intravenous access is not possible. Patients should be monitored after treatment for adverse effects, including excessive sedation, ataxia and nasal irritation. As there is only one clinical trial directly comparing IN-MDZ and R-DZP in dogs, the strength of the evidence is limited.

## Summary of the evidence

**Charalambous et al. (2017):** 

Population	Client-owned dogs with idiopathic or structural epilepsy presenting to a participating veterinary centre (across Europe) in status epilepticus (generalised or focal). Exclusion Criteria: Reactive seizures due to metabolic or toxic cause. Dogs that had received any drugs before 5 minutes of continuous seizure activity had passed.
Sample Size	35 dogs (initially 38 dogs but 3 were subsequently excluded as their seizure diagnosis did not meet inclusion criteria): 33 presented with generalised tonic/clonic seizures. 2 presented with focal seizures.
Intervention Details	Status epilepticus was defined as continuous seizure activity lasting more than 5 minutes, or more than 2 discrete seizures with incomplete recovery of consciousness between seizures. In all cases, the attending clinician prepared and administered the treatment: Dogs seizuring on admission were assumed to be in status epilepticus (over 5-minute duration) and treatment was administered immediately by the clinician. Inpatients seizuring were monitored by the nurse or student in the ward for 3 minutes before the clinician was contacted; treatment was administered on arrival of the clinician. Dogs were assigned into treatment groups (intranasal midazolam (IN-MDZ) or rectal diazepam (R-DZP)) using randomised sealed envelopes. Treatment: IN-MDZ treatment (n = 20). 2 mg/kg midazolam injectable solution (5 mg/ml) administered intranasally (IN) via a mucosal atomisation device (MAD). Doses of >1 ml were divided between both nostrils to reduce drug outflow. R-DZP treatment (n = 15). 1 mg/kg diazepam injectable solution (5 mg/ml) per rectum via a needleless syringe applied as deep as possible into the rectum. All dogs were hospitalised in an ICU or equivalent environment for continuous observation and monitoring for at least 24 hours after drug administration. For unsuccessful cases, 0.5–1.0 mg/kg diazepam was administered IV.
Study design	Multicentre randomised open-label clinical trial.
Outcome studied	Seizure cessation was defined as the termination and absence of visible seizure-related motor activity. Primary outcomes: Seizure cessation time – time between drug administration and seizure cessation. Seizure relapse time – time between seizure cessation and onset of next seizure. Successful treatment was defined as: Seizure cessation time < 5 minutes. Seizure relapse time > 10 minutes. Where treatment was unsuccessful and the patient required IV diazepam, time for seizure cessation after administration was recorded. Additional Measurements: ‘Call to doctor’ time – time taken for clinician to arrive. ‘Doctor to drug time’ – time taken for preparation and administration of assigned drug (IN-MDZ / R-DZP). Patient Outcomes: Objective measurements of heart rate and rhythm, respiratory rate and pattern, blood pressure (via doppler) and oxygen saturation (pulse oximetry) assessed at 10 and 60 minutes after treatment. Subjective analysis of sedation and ataxia at 0, 10, and 60 minutes after treatment using a 9 cm visual analogue scale. Scores from 0–9 cm = ‘mild’, 3.0–5.9 cm = ‘moderate’, 6.0–9.0 = ‘severe’. Any additional unusual or adverse effects were recorded. Subjective ‘ease of administration’ was also recorded by the clinician.
Main findings (relevant to PICO question)	IN-MDZ had a significantly greater ‘success’ rate (seizure cessation < 5 minutes, no relapse < 10 minutes) than R-DZP (P = 0.0059), especially for idiopathic epilepsy (P = 0.018). 70% (14/20) dogs successful with IN-MDZ (95% CI: 48–85%) vs 20% successful (3/15) dogs with R-DZP (95% CI 6.6–43%). IN-MDZ caused more rapid cessation of seizure activity (median 47 seconds, range 6–280 seconds) than R-DZP (median 214 seconds, range 204–290 seconds). IN-MDZ prevented relapse in 21% (3/14) successful cases, whilst all successful R-DZP cases relapsed (3/3). Time before relapse was longer for IN-MDZ cases (median 15 minutes, range 10–19 minutes) than R-DZP cases (median 10.8 minutes, range 10.6–2 minutes). ‘Doctor-to-drug’ for IN-MDZ was longer than that for R-DZP. IN-MDZ median 29 seconds (14–185); R-DZP median 16 seconds (8–38). Severe sedation and ataxia were reported for all dogs in both treatment groups, and this appeared to worsen over the initial 60-minute monitoring period. No patients in either group had signs of respiratory or cardiovascular compromise. Sneezing episodes were reported in 7/20 (35%) of patients treated with IN-MDZ; no such signs were seen in R-DZP patients. Difficulties in administration were reported for 10% (2/20) IN-MDZ cases, and 13% (2/15) R-DZP cases. The authors concluded that IN-MDZ was a quick, safe and effective first-line medication for controlling canine status epilepticus, and may be superior to R-DZP, especially in cases of idiopathic epilepsy. Overall findings support the use of IN-MDZ for treating seizures in emergency patients where IV access is not achievable.
Limitations	The minimum sample size for 80% power was calculated as 36 per group (total 72); there were a total of 35 dogs included in the study, with an unequal distribution of patients between groups, despite randomisation, which limits the power of the study and reduces validity of the results. Few patients (n = 2) were reported as having focal seizures, and these were both allocated to the IN-MDZ group. Therefore, this study cannot be used to compare the efficacy of IN-MDZ and R-DZP for controlling focal seizures in canine patients. The small sample size limits the ability to detect rare adverse effects. P-values are only reported for comparison of ‘successful treatment’ (defined as seizure cessation within 5 minutes and no relapse within 10 minutes) between groups. Statistical tests for differences between individual measured outcomes such as ‘doctor-to-drug time’ and incidence of adverse effects are not reported, so it is unclear whether the difference between treatment groups is significant at the individual outcome level. Seizure cessation was defined as ‘the termination and absence of visible seizure-related motor activity’, relying on subjective interpretation by the attending clinician. Continued micromotor activity or focal seizures may have been missed, leading to incorrect reporting of seizure cessation. Post-ictal behavioural abnormalities may have been reported incorrectly as continued seizure activity by some clinicians Clinicians were not blinded to the treatment that each patient had received, potentially leading to observer bias when defining seizure cessation, scoring sedation and ataxia and reporting adverse effects. For example, sneezing might have occurred in the R-DZP group but not have been seen as clinically ‘relevant’ and therefore ignored, whereas in the IN-MDZ group this would be more likely to be recorded as an adverse event. Duration of seizure activity before treatment was administered may have varied significantly between patients; prolonged seizures may be refractory or resistant to treatment, reducing the likelihood of success. Status epilepticus was assumed for all patients arriving to the hospital still seizuring. However, as the seizures were unwitnessed by the clinicians it is unclear if these patients truly met the authors’ definition of status epilepticus, and seizure duration may have been significantly longer than the assumed 5 minutes. Patients already in the hospital were monitored by either a nurse or veterinary student in the ward, and the clinician alerted after 3 minutes of observed seizure activity; however, there may still have been discrepancy between seizure onset and the time for this to be noticed. As this was a multi-centre study, different clinicians and support staff would have been involved in each case. The report does not indicate which cases attended each clinic, so this cannot be statistically accounted for. Despite use of a standardised protocol, there is likely to be variation between clinicians and centres, especially for subjective measurements. According to the protocol, the clinician was responsible for preparing and administering the treatment in all cases; however, timing and recording was performed by various support staff – nurses, students and technicians – across different centres, increasing risk of human error and observer bias. Use of a visual analogue scale to measure sedation and ataxia introduces subjectivity and potential observer bias, particularly as the researchers were not blinded to the treatment received. IN-MDZ was administered via a human mucosal atomisation device; results cannot be generalised to situations where IN-MDZ is administered without this device. As suggested by the authors, ‘the use of the nasal mucosal atomisation device for IN-MDZ administration may have contributed to increased efficacy’ and ease of use of this treatment. All treatment occurred in a hospital environment, so the results cannot be generalised to other veterinary environments.

Table note...

## Appraisal, application and reflection

Status epilepticus is a life-threatening medical emergency requiring rapid and effective seizure control to reduce morbidity and mortality (Platt, 2014b; Charalambous et al., 2021; Charalambous et al., 2022). Benzodiazepines are frequently used as first-line medications in both human and canine patients due to their high potency and rapid onset of action (Charalambous, et al., 2021). These can be administered via various routes, with the intravenous (IV) route often preferred as it provides direct access to the circulation and 100% drug bioavailability (Schwartz, et al., 2013). However, in emergency situations establishing IV access may be difficult, requiring the use of alternative administration routes (Schwartz et al., 2013; Davis et al., 2021). Furthermore, these routes may be used outside of the clinical environment, including at home (Mula, 2017).

Rectal diazepam (R-DZP) is a commonplace first-line treatment for seizures, including status epilepticus, in both human and veterinary medicine (Platt, 2014a). However, rectal drug administration can be challenging in actively seizuring patients (De Haan et al., 2010). Furthermore, bioavailability of rectally administered drugs is variable, with limited maximum plasma concentrations and slow onset of action (Eagleson et al., 2012; Schwartz et al., 2013). Intranasal (IN) drug administration allows rapid drug absorption into the systemic circulation close to the brain, avoiding first-pass hepatic metabolism and encouraging accumulation of the drug within neurological tissue (Charalambous, 2018). In human patients, the IN route is more convenient and socially acceptable than rectal administration (Eagleson et al., 2012). A meta-analysis of studies investigating benzodiazepines for control of status epilepticus in human paediatric patients concluded that midazolam was superior for obtaining seizure control when compared to diazepam, with non-IV midazolam achieving the same efficacy as IV diazepam (McMullan et al., 2010). Furthermore, midazolam solutions are less irritant than diazepam solutions, reducing risk of irritation when administered intranasally (Schwartz et al., 2013; Platt, 2014a).

This Knowledge Summary seeks to appraise the evidence for use of intranasal midazolam (IN-MDZ) as an effective and safe alternative to R-DZP to control status epilepticus in canine patients. A literature search found a mixture of evidence, including two veterinary multi-centre open-label randomised clinical trials, a human open-label randomised clinical trial, and a human double-blinded placebo-controlled clinical trial. Few primary veterinary studies were found, indicating a need for further research to allow confident conclusions to be made. Only the study by Charalambous et al. (2017) directly related to all aspects of the PICO question, limiting the strengths of the conclusions that can be drawn, especially as the study was open-label and had a small sample size, reducing statistical power (Burmeister & Aitken, 2012). A follow-up study by Charalambous et al. (2019) compared IN-MDZ and IV-MDZ, further supporting the use of IN-MDZ in canine patients. However, as this paper did not directly compare IN-MDZ and R-DZP it did not meet the inclusion criteria for critical analysis. Whilst the conclusions from human clinical trials (Javadzadeh et al., 2012; Detyniecki et al., 2019) support those of the veterinary studies, care must be taken when applying results across species due to differences in pharmacokinetics (Potschka, et al. 2013; Uriarte & Maestro Saiz, 2016). A recent survey by Kähn et al. (2023) investigated dog owner perspectives on various out-of-hospital seizure control medications, including R-DZP and IN-MDZ. However, as this was not a clinical trial it does not meet the criteria for critical analysis.

When directly comparing IN-MDZ and R-DZP in canine patients, Charalambous et al. (2017) found that IN-MDZ was significantly more successful than R-DZP in achieving rapid seizure control and preventing further seizure activity. In this clinical trial, the time for administration of IN-MDZ was longer than for R-DZP, yet this did not affect overall drug efficacy. As explained by Kähn, et al. (2023), R-DZP is often administered from a pre-drawn syringe or suppository, whereas IN-MDZ administration requires several additional steps before the drug can be administered. Furthermore, unfamiliarity with the nasal mucosal atomisation device used for IN drug administration may increase the time taken for drug administration, as suggested by the authors of a similar human study (De Haan et al., 2010); trials performed after a period of regular use or training would allow a more accurate comparison. Despite being a multi-centre study, this investigation (Charalambous et al., 2017) had a small overall sample size and therefore limited statistical power to identify true differences between groups (Burmeister & Aitken, 2012). Furthermore, as noted by Packer et al. (2015), interpretation of seizure activity and paroxysmal events can be significantly different between clinicians; patients presenting to different hospitals may have been classified (and therefore treated) differently depending on clinician interpretation.

Additional papers indirectly supporting the findings of Charalambous et al. (2017) did not address all aspects of the PICO question and therefore did not meet the inclusion criteria for critical analysis. Relevant studies included a comparison of IN and intravenous midazolam (IV-MDZ) in canine patients in status epilepticus (Charalambous et al., 2019). Two human studies were also found; one was an open-label clinical trial comparing the efficacy of IN-MDZ and intravenous diazepam in controlling status epilepticus (Javadzadeh et al., 2012), and the other was a double-blinded randomised placebo controlled trial investigating the safety and efficacy of IN-MDZ for controlling cluster seizures (Detyniecki et al., 2019).

Detyniecki et al. (2019) investigated IN-MDZ as a treatment for cluster seizures in human patients, reporting this to be significantly more effective than a placebo for seizure control and preventing recurrence, with tolerable safety margins. In human patients, cluster seizures may progress to status epilepticus, but this has not been sufficiently investigated in veterinary patients (Platt, 2014b). Therefore, the results from Detyniecki et al. (2019) cannot be directly applied to canine patients or used to answer the PICO question.

When comparing IN-MDZ to intravenous midazolam (IV-MDZ) at the same dose in canine patients, Charalambous et al. (2019) found no significant difference in efficacy between treatment groups. Seizure cessation time was shorter in the IN-MDZ group, and this difference became statistically significant when accounting for the time taken to obtain IV access. These conclusions contrast with those of Javadzadeh et al. (2012), who compared IN-MDZ and IV-DZP in human children; intravenous diazepam (IV-DZP) achieved more rapid seizure control than IN-MDZ once the drug had been administered. However, obtaining IV access delayed drug administration, increasing the overall duration before seizure control was obtained in the IV-DZP group. Similarly, Mahmoudian & Mohammadi Zadeh (2004) found no significant difference in effectiveness between IN-MDZ and IV-DZP for controlling seizure activity in human patients. However, both of these studies found that seizure control was more rapid with IV-DZP than IN-MDZ once IV access was established, suggesting that this may be a preferred route of administration for patients with an IV catheter already in situ (Mahmoudian & Mohammadi Zadeh, 2004; Javadzadeh et al., 2012). This is clinically significant, as the prognosis for status epilepticus is time-dependent, with longer seizure duration associated with development of treatment-resistance and poor clinical outcomes (Charalambous, et al., 2019; Messahel et al., 2022). Therefore, finding an effective treatment option which can be rapidly administered is essential; these studies suggest that IN-MDZ better meets these criteria than trying to obtain IV access when patients are experiencing seizure activity prior to IV access being established. Due to species differences between humans and canine patients, the results from human studies cannot directly be extrapolated to the veterinary population. However, whilst the Mahmoudian & Mohammadi Zadeh (2004) and Javazadeh et al. (2012) studies do not directly compare IN-MDZ to R-DZP, their conclusions indirectly support those of Charalambous, et al., 2017; as IV drug administration is generally considered superior to rectal administration due to more rapid achievement of higher peak plasma concentrations (Eagleson et al., 2012), it is reasonable to extrapolate from these results that IN-MDZ is more clinically useful than rectal drug administration when IV access is not possible.

In many studies (Charalambous et al., 2017; Charalambous et al., 2019; Detyniecki et al., 2019), IN-MDZ was administered via a mucosal atomisation device; these devices create a fine mist of small particles, enhancing drug absorption, andtherefore bioavailability. Javadzadeh et al. (2012) did not use such a device in their study, instead administering IN-MDZ to human patients via a syringe. Detyniecki et al. (2019) used a novel combination product designed as a single-dose nasal spray for human patients; however, as the authors do not report the time taken for seizure control when using this product, results from this study cannot be directly compared to those of Javadzadeh et al. (2012) to determine the effect of the device. However, the canine studies (Charalambous et al., 2017; Charalambous et al., 2019) demonstrated faster times for seizure cessation with IN-MDZ administered via the mucosal atomisation device than those reported by Javadzadeh et al. (2012), where IN-MDZ was administered directly via a syringe, suggesting the device may improve efficacy of IN-MDZ. However, no study directly comapring the efficacy of IN-MDZ administered via a syringe to IN-MDZ administered via an atomisation device in canine patients was found; further research is warranted to determine the impact of such a device. Furthermore, as noted by Charalambous et al. (2019), no veterinary-specific devices are yet available; species- and breed-specific devices designed for veterinary patients would likely enhance the efficacy of IN-MDZ.

In all the reviewed studies, definitions of seizure activity and control were based on clinician interpretation of clinical signs rather than objective measurements of brain electrical activity. This is particularly significant in open-label trials, where observer bias may have influenced recording and reporting of data. Theoretically, double-blinded clinical trials relying on self-reporting by patients or their caregivers have a lower risk of bias; however, caregiver recognition, and reporting of seizure activity, and treatment efficacy may be unreliable (Akman et al., 2009). Furthermore, the data recorded by caregivers may be less accurate than that recorded by scientific and medical professionals, especially during a stressful situation (such as witnessing a seizure and administering a novel treatment (Cushner-Weinstein et al., 2008)).

IN-MDZ appears to be safe in human and veterinary patients; no significant adverse effects were reported in either of the veterinary trials (Charalambous et al., 2017; Charalambous et al., 2019) or in human patients by Javadzadeh et al. (2012). However, these studies were underpowered to detect rare adverse events, and single point in time trials cannot detect delayed adverse effects or those associated with chronic administration (Edwards et al., 1999). The double-blinded placebo-controlled trial by Detyniecki, et al. (2019) revealed a higher incidence of adverse effects in human patients receiving IN-MDZ when compared to a placebo group, including severe adverse reactions in 3% of IN-MDZ treated patients; no severe adverse reactions were reported in the placebo group. However, the authors did not report whether the difference in incidence of adverse reactions between the treatment groups was statistically significant (Detyniecki et al., 2019). In canine patients, common adverse effects appear to be related to benzodiazepine administration – including sedation, ataxia, and dysphoria – regardless of administration route (Charalambous, et al., 2017; Charalambous, et al., 2019). IN-MDZ was also associated with brief sneezing episodes in canine patients, suggesting nasal irritation (Charalambous et al., 2017; Charalambous et al., 2019). Human patients receiving intranasal administration of a placebo also reported nasal discomfort (Detyniecki et al., 2019), suggesting that this adverse effect may be related to the route of administration rather than the drug itself. Survey findings from Kähn et al. (2023) suggest that IN-MDZ is more successful than R-DZP for controlling seizures in dogs at home, with earlier seizure termination and less frequent repeat dosing for seizure control. Furthermore, the survey found owner compliance and satisfaction to be higher with IN-MDZ than R-DZP, suggesting this may be a preferable medication for recommendation for at-home use in canine patients with epilepsy.

Overall, there is little direct evidence that IN-MDZ is superior to R-DZP for controlling status epilepticus in canine patients, but extrapolation of results from human clinical trials and studies comparing IN-MDZ to other routes of administration suggest that it may be a superior first-line treatment option. Further large-scale randomised controlled trials in canine patients are needed to provide a stronger evidence base.

## Methodology

**Search strategy:** 

	Search strategy
Databases searched and dates covered	CABI Direct via CAB Abstracts (2012 to May 2024) PubMed via NIH (2012 to May 2024) Wiley Online Library (2012 to May 2024) PMC via NIH (2012 to May 2024)
Search terms	CABI Direct: (Canine OR dog) (Seizure OR Epilep*) (intranasal midazolam) ‘Rectal Diazepam’ 1 AND 2 AND 3 AND 4 (4) 1 AND 2 AND 3 (5) PubMed: (Canine OR dog) (Seizure OR Epilep* OR ‘status epilepticus’) (‘intranasal midazolam’) ‘Rectal diazepam’ 1 AND 2 AND 3 AND 4 (2) 1 AND 2 AND 3 (4) Wiley: (Canine OR dog) (Seizure OR Epilep*) ‘intranasal midazolam’ ‘Rectal diazepam’ 1 AND 2 AND 3 AND 4 (1) 1 AND 2 AND 3 (2) PMC: (Canine OR dog) ‘status epilepticus’ ‘Intranasal midazolam’ ‘Rectal Diazepam’ 1 AND 2 AND 3 AND 4 (46) 1 AND 2 AND 3 (68)
Dates searches performed:	04 May 2024

Table note placeholder

**Exclusion / inclusion criteria:** 

Exclusion	Articles not relevant to the PICO. Non-primary literature: review articles / meta-analyses / clinical guidelines / conference proceedings / expert opinion articles / book chapters. Articles that were not fully accessible via OpenAthens. Articles not available in English. Duplicates.
Inclusion	Comparative studies / clinical trials investigating the safety and efficacy of IN-MDZ for seizure control in caninepatients. Studies published between 2012–2024.

Table note placeholder

**Search outcome:** 

Database	Number of results	Excluded – primary studies not relevant to PICO	Excluded –non- primary literature	Excluded – not accessible / abstract only	Excluded – duplicates	Total relevant papers
CABI Direct	9	1	2	1	4	1
PubMed	6	3	0	0	3	0
Wiley	3	1	0	0	2	0
PMC	114	54	59	0	1	0
Total relevant papers when duplicates removed						1

Table note placeholder

## Acknowledgements

The author would like to thank Evelyn O’Byrne for her help and support during the writing process.

## ORCiD

Flora Foxx: https://orcid.org/0000-0002-8729-4982

## Conflict of interest

The author declares no conflicts of interest.

## Disclaimer

Knowledge Summaries are a peer-reviewed article type which aims to answer a clinical question based on the best available current evidence. It does not override the responsibility of the practitioner. Informed decisions should be made by considering such factors as individual clinical expertise and judgement along with patient’s circumstances and owners’ values. Knowledge Summaries are a resource to help inform and any opinions expressed within the Knowledge Summaries are the author’s own and do not necessarily reflect the view of the RCVS Knowledge. Authors are responsible for the accuracy of the content. While the Editor and Publisher believe that all content herein are in accord with current recommendations and practice at the time of publication, they accept no legal responsibility for any errors or omissions, and make no warranty, express or implied, with respect to material contained within. For further information please refer to our Terms of Use.
